# New Approach to Detect and Isolate Rhamnogalacturonan-II in *Arabidopsis thaliana* Seed Mucilage

**DOI:** 10.21769/BioProtoc.5433

**Published:** 2025-09-05

**Authors:** Dayan Sanhueza, Susana Saez-Aguayo

**Affiliations:** Laboratorio Mucilab, Centro de Biotecnología Vegetal, Facultad de Ciencias de la Vida, Universidad Andrés Bello, Santiago, Chile

**Keywords:** Rhamnogalacturonan-II, Size exclusion chromatography (SEC), *Arabidopsis thaliana*, Seed mucilage, Pectin isolation, Uronic acid quantification, Polyacrylamide gel electrophoresis (PAGE).

## Abstract

Rhamnogalacturonan-II (RG-II) is one of the least studied domains of pectin, primarily due to its low abundance, the lack of reliable antibodies, and the complexity of its structure. The present study builds upon existing protocols and procedures used to analyse RG-II in tissues where it is more abundant, combining and adapting them for the isolation of RG-II from *Arabidopsis* seed mucilage—a structure previously thought to lack RG-II. By applying these adapted methods, we first confirmed the presence of RG-II in seed mucilage and subsequently succeeded in isolating it from a tissue where it is typically present in low abundance, thereby enabling future studies on this previously overlooked component.

Key features

• An efficient RG-II isolation protocol offers the opportunity to simplify and deepen the study of RG-II.

• It provides reliable yields.

• The protocol allows analysis of RG-II synthesis or structural changes affecting dimerisation in mutant lines using *Arabidopsis* seed mucilage.

## Graphical overview


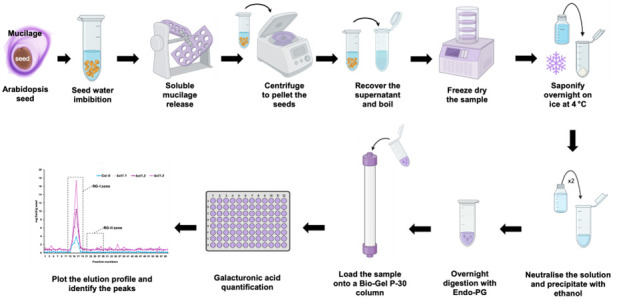


## Background

The mucilage of the *Arabidopsis* seed coat is a gel-like structure produced by specialised epidermal cells [1]. It contains all major polysaccharide groups found in plant cell walls, with pectins comprising 90%–95% of its composition, alongside smaller amounts of other components. The mucilage is predominantly composed of rhamnogalacturonan-I (RG-I), with lesser quantities of homogalacturonan (HG), cellulose, galactoglucomannans, xylans, xyloglucans, and arabinogalactan proteins (AGPs) [2–5]. Although rhamnogalacturonan-II (RG-II) has been proposed to contribute to mucilage organisation, its presence within the mucilage layers remains largely unexplored [5]. Research on RG-II has often been limited due to its low abundance and the absence of reliable analytical tools. Unlike what occurs with plant tissues, where the amount of material is greater and RG-II is more abundant compared to mucilage, it is not possible in this case to directly visualise RG-II without prior enrichment of this domain. In this report, isolated mucilage was processed to evaluate the feasibility of applying protocols previously developed for RG-II-rich tissues [6] to detect and isolate this domain from *Arabidopsis* seed mucilage. We optimised the amount of dry seed required to extract and process the mucilage needed for size-exclusion chromatography, and successfully isolated RG-II despite its low abundance. By taking advantage of the distinct sizes of RG-I and RG-II following enzymatic digestion with endo-polygalacturonase, we were able to specifically isolate RG-II by determining the elution profile through galacturonic acid quantification [7]. To confirm that the isolated domain was indeed RG-II, we monomerised the dimeric form and conducted a dimerisation assay [6], validating the identity of the recovered polymer. This approach provides a valuable tool for investigating polysaccharide biosynthesis—particularly that of RG-II—using mutants with defects in mucilage structure or function.

## Materials and reagents

**Biological materials**

1. Seeds of *Arabidopsis thaliana* Col-0

2. Seeds of *Arabidopsis thaliana bxl1* (mutant)

**Reagents**

1. MilliQ water

2. Pyridine (Sigma-Aldrich, CAS: 110-86-1)

3. Acetic acid, glacial (Merck, CAS: 64-19-7)

4. Endo-polygalacturonase (Megazyme, CAS: 9032-75-1)

5. Ammonium persulfate (Thermo Scientific, CAS: 7727-54-0)

6. Tris-base (Merck, CAS: 77-86-1)

7. Hydrochloric acid (HCl) (Merck, CAS: 7647-01-0)

8. Tetramethylethylenediamine (TEMED) (Thermo Scientific, catalog number: 17919)

9. Ethanol (Merck, CAS: 64-17-5)

10. Sodium thiosulphate (Sigma-Aldrich, CAS: 7772-98-7)

11. Silver nitrate (Merck, CAS: 7761-88-8)

12. Formaldehyde (Winkler, CAS: 50-00-0)

13. Sodium carbonate (Na_2_CO_3_) (Sigma-Aldrich, CAS: 497-19-8)

14. Sulfuric acid (Merck, CAS: 7664-93-9)

15. Sodium hydroxide (NaOH) (Sigma-Aldrich, CAS: 1310-73-2)

16. 3-Hydroxybiphenyl (Sigma-Aldrich, CAS: 580-51-8)

17. Chlorobutanol (Sigma-Aldrich, CAS: 6001-64-5)

18. Sodium tetraborate decahydrate (Borax decahydrate) (Sigma-Aldrich, CAS: 1303-96-4)

19. D-(+)-Galacturonic acid monohydrate (GalA) (Merck, CAS: 91510-62-2)

20. Lead nitrate (Merck, CAS: 10099-74-8)

21. Glycine (Merck, CAS: 56-40-06)

22. Acrylamide/Bis acrylamide (29:1) 40% (Bio-Rad, catalog number 1610147)

23. Bromophenol blue (Merck, CAS: 115-39-9)

**Solutions**

1. 0.5 M Na_2_CO_3_ (see Recipes)

2. Pyridine:acetic acid:water (PyAW, 1:10:200 v/v/v), 0.25% chlorobutanol (see Recipes)

3. Pyridine:acetic acid:water (PyAW, 1:1:98 v/v/v), 0.25% chlorobutanol (see Recipes)

4. Solution A (see Recipes)

5. Solution B (see Recipes)

6. Galacturonic acid stock solution (see Recipes)

7. Tris-HCl 1.5 M, pH 8.8 (see Recipes)

8. Ammonium persulfate 10% (APS) (see Recipes)

9. Polyacrylamide gel 26.4% (see Recipes)

10. Running buffer for PAGE (see Recipes)

11. Loading buffer (see Recipes)

12. Fixing solution (see Recipes)

13. 400 µM sodium thiosulfate solution (see Recipes)

14. 6 mM silver nitrate and 10 mM formaldehyde solution (see Recipes)

15. Developing solution (see Recipes)

16. Stopping solution (see Recipes)

**Recipes**

**1. 0.5 M Na_2_CO**_3_

Add 5.3 g of Na_2_CO_3_ to 100 mL of MilliQ water.

**2. Pyridine:acetic acid:water (PyAW, 1:10:200 v/v/v), 0.25% chlorobutanol**

Example for 100 mL:

**Table:** 

Reagent	Quantity/Volume
Pyridine	47 μL
Acetic acid	4.74 mL
Water	9.47 mL
Chlorobutanol	0.25 g

**3. Pyridine:acetic acid:water (PyAW, 1:1:98 v/v/v), 0.25% chlorobutanol**

Example for 1 L:

**Table:** 

Reagent	Quantity/Volume
Pyridine	10 mL
Acetic acid	10 mL
Water	980 mL
Chlorobutanol	2.5 g

**4. Solution A**

Add 0.5 g of Borax decahydrate to 100 mL of sulfuric acid.

**5. Solution B**

Add 0.4 g of NaOH to 10 mL of water; then, add 0.015 g of 3-hydroxybiphenyl.

**6. Galacturonic acid stock solution**

Prepare a 0.1 mg/mL stock solution of galacturonic acid (GalA) by dissolving 1 mg of GalA in 10 mL of MilliQ water.

**7. Tris-HCl 1.5 M, pH 8.8**

To prepare 1 L, weigh 181.65 g of Tris base and dissolve it in 700 mL of MilliQ water. Add 31 mL of hydrochloric acid, and then adjust the pH to the desired value using 1 M HCl. Finally, bring the volume up to 1 L with MilliQ water.

**8. Ammonium persulfate 10% (APS)**

To prepare 10 mL, add 1 g of ammonium persulfate to 10 mL of MilliQ water.

**9. Polyacrylamide gel 26.4%**

Example for 5 mL (one mini gel, using 0.75 mm spacers):

**Table:** 

Reagent	Quantity
Tris base 1.5 M pH 8.8	834 μL
Acrylamide/Bis acrylamide (29:1) 40%	3.33 mL
APS	46.7 μL
TEMED	3.9 μL
Water	834 μL

**10. Running buffer for PAGE**

Example for 1 L (10×):

**Table:** 

Reagent	Quantity
Tris base	60.57 g
Glycine	28.53 g

**11. Loading buffer**

To prepare 20 mL of buffer, dissolve 1.53 g of Tris in 7 mL of water, adjust the pH to 6.8 with 1 M HCl, and make up to 10 mL with water. Then, add 0.05 g of bromophenol blue and 10 mL of glycerol.

**12. Fixing solution**

Example for 1 L:

**Table:** 

Reagent	Quantity/Volume
Ethanol	400 mL
Acetic acid	100 mL
Water	500 mL

**13. 400 µM solution of sodium thiosulfate**

Add 63.2 mg of sodium thiosulfate to 1 L water.

**14. 6 mM silver nitrate and 10 mM formaldehyde solution**

Add 0.102 g of silver nitrate and 75.2 μL of 37% formaldehyde to 100 mL of water.

*Note: This solution must be freshly prepared.*

**15. Developing solution**

Add 29.68 g of sodium carbonate, 20 mL of 400 µM sodium thiosulfate solution (Recipe 13), and 4.8 mL of 37% formaldehyde to 975.2 mL of water.

**16. Stopping solution**

Add 40 g of Tris to 980 mL of water, then complete to 1 L with 20 mL of glacial acetic acid.

**Laboratory supplies**

1. Bio-Gel P-30 (Bio-Rad, catalog number: 1504154)

2. 96-well plate (Nest, catalog number: 514201)

3. Epoch 96-well plate absorbance reader (Agilent BioTek Epoch Microplate Spectrophotometer, catalog number: 11-120-572)

## Equipment

1. Pipettes (Corning, model: Lambda^TM^ Plus Single, catalog numbers: 4070, 4082, and 4075)

2. Chromatography column (Bio-Rad, model: Econo-Column^®^, catalog number: 7372576)

3. Electrophoresis system (Bio-Rad, model: Mini-PROTEAN^®^ Tetra Cell, catalog number: 165-8004)

4. Speed-vac (Eppendorf, model: Concentrator plus, catalog number: 5305000568)

## Procedure

**A. Sample preparation for the chromatography column**

1. Weigh approximately 50 mg of dry seeds.

2. Isolate soluble mucilage by incubating the seeds in 4 mL of MilliQ water for 2 h with agitation.

3. Centrifuge the sample at 3,500× *g* for 5 min.

4. Recover the supernatant, immediately boil it at 100 °C for 5 min, and immediately store it at -20 °C.

5. Freeze-dry the sample.

6. Resuspend the dried mucilage in 500 μL of 0.5 M Na_2_CO_3_ and incubate overnight at 4 °C.

7. Neutralise the Na_2_CO_3_ with approximately 50 μL of glacial acetic acid, monitoring the reaction by observing the cessation of fizzing.

*Note: The reaction between Na_2_CO_3_ and acetic acid (CH_3_COOH) is an acid–base reaction that produces sodium acetate, water, and carbon dioxide, which is released as a gas, visible as bubbling or fizzing.*

8. Precipitate the mucilage by adding two volumes (1.1 mL) of absolute ethanol.

9. Pellet the mucilage by centrifuging at 3,500× *g* for 5 min.

*Note: Steps 4 to 9 can be visualised in the Video 1.*

10. Rinse the pellet twice with 80% ethanol, followed by a final rinse with absolute ethanol.

*Note: Rinsing is performed by vortexing the sample.*

11. Resuspend the mucilage in 2 mL of PyAW, 1:10:200 v/v/v containing 0.25% chlorobutanol, and digest overnight at 25 °C with agitation using 1.1 U endo-polygalacturonase.

12. Centrifuge the sample at 3,500× *g* for 5 min to pellet any undigested material.

13. Load the entire supernatant (2 mL) remaining after centrifugation onto a Bio-Gel P-30 column (2.5 × 57 cm). Set the flow rate of the column to 1 mL per min.

14. Use PyAW, 1:1:98 v/v/v containing 0.25% chlorobutanol as the eluent.

15. Discard the first 10 mL of eluent, then collect 60 fractions of 5 mL each.

**Video 1.**
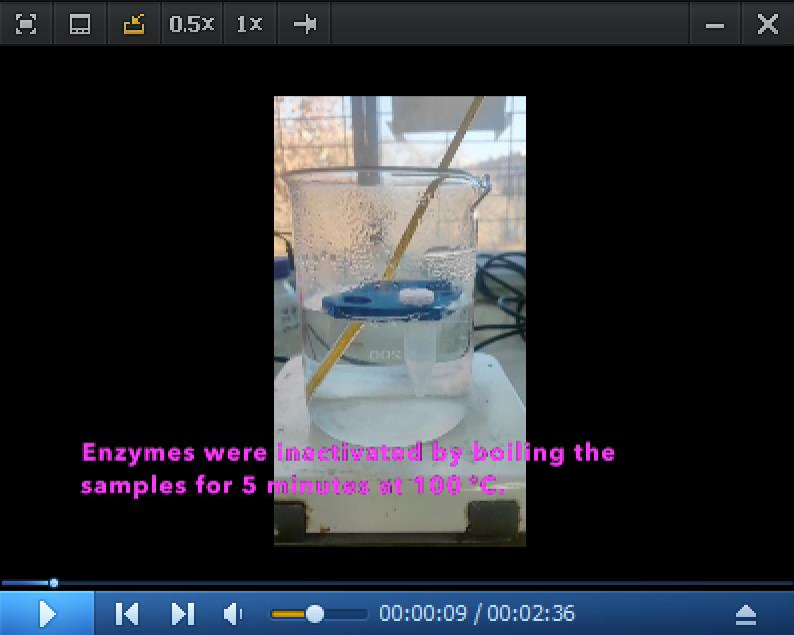
Key steps in mucilage processing

**B. Uronic acid quantification**

1. Add 20 μL of the sample fraction to 100 μL of solution A.

2. Mix the solution by vortexing, then incubate at 100 °C for 5 min.

3. Transfer the entire volume (120 μL) to a 96-well plate.

4. Measure the absorbance at 520 nm.

5. Add 2 μL of solution B and mix thoroughly by pipetting.

6. Measure the absorbance again at 520 nm.

7. Plot a graph of the amount of GalA (µg) against the fraction number.

8. The concentration is determined using the calibration curve described in Table 1.

**Figure 1.**
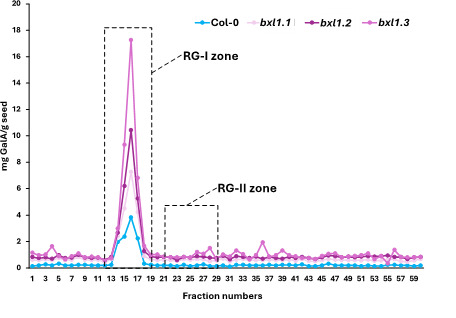
Uronic acid quantification. As expected for a mucilage sample, the majority of the material corresponds to rhamnogalacturonan-I (RG-I) (between fractions 11 and 19). Based on our experience with other types of samples, rhamnogalacturonan-II (RG-II) elutes between fractions 21 and 29. Therefore, we proceed to concentrate these fractions using a speed vacuum.

*Note: This enables identification of the peak corresponding to the elution zone of RG-II (Figure 1).*

**C. Galacturonic acid standard curve**

1. Prepare 80 μL of each GalA concentration in the calibration series.

**Table 1.** Calibration series of GalA concentration

GalA amount (µg)	GalA stock solution (µL)	MilliQ water (µL)
0	0	20
0.5	5	15
1	10	10
1.5	15	5
2	20	0

2. Repeat steps B1–7, but replace samples for each GalA concentration.

*Note: We recommend using three replicates.*

**D. Sample concentration**

1. Pool the fractions corresponding to tubes 21–29 and dry them using a speed vac at room temperature.

2. Once dry, resuspend the sample in 200 μL of MilliQ water.

3. Quantify the GalA concentration by repeating steps B1–7.

4. Prepare a 0.1 µg/μL solution.

**E. Polyacrylamide gel electrophoresis (PAGE)**

1. Cast the PAGE gel following the instructions provided.

2. Mix 8 µL of the 0.1 μg/μL RG-II solution with 2 μL of loading buffer and load the sample into the gel.

3. Run the gel at 200 V for 75 min.

4. Incubate for 30 min in the fixing solution.

5. Wash three times, 1 min each, with MilliQ water.

6. Incubate for 1 min in the 400 μM sodium thiosulfate solution.

7. Wash three times, 1 min each, with MilliQ water.

8. Incubate for 20 min in the solution containing 6 mM silver nitrate and 10 mM formaldehyde.

9. Wash twice with MilliQ water, 20 s each.

10. Incubate with the developing solution for 2–10 min or until bands become visible.

11. Stop the reaction by adding the stopping solution.

## Data analysis

**Figure 2.**
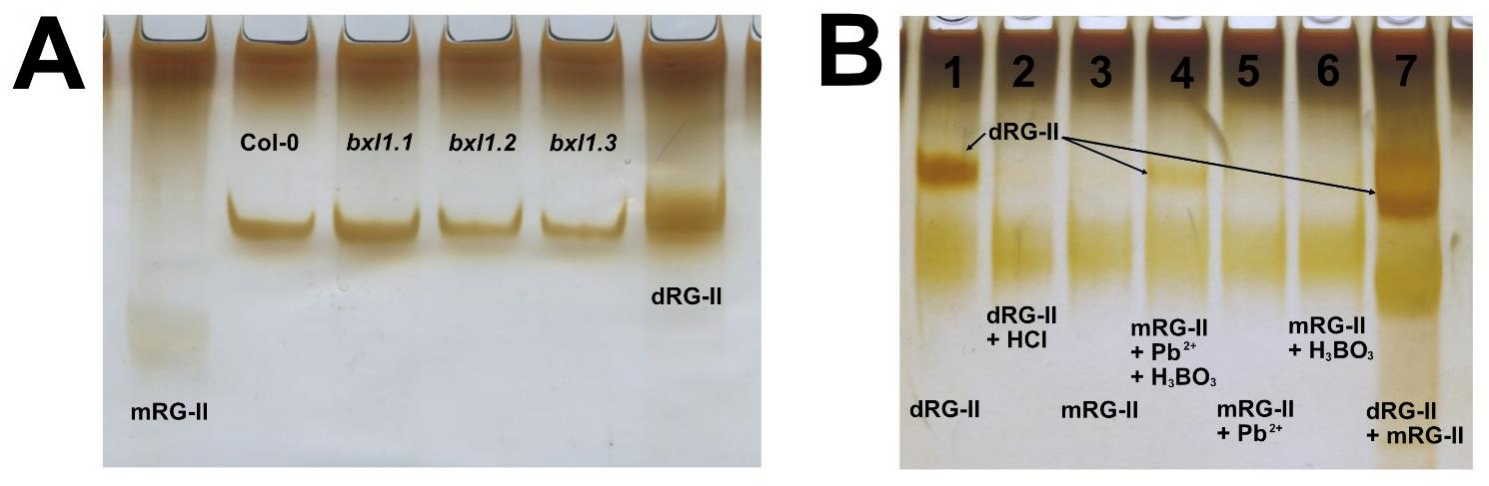
PAGE analysis of isolated RG-II domains. (A) Using the protocol described by Chormova et al. [6], the isolated RG-II can be visualised by electrophoresis. The figure presents RG-II isolated from *Arabidopsis* Col-0 and three different allelic mutants of the *AtBXL1* gene. As molecular markers, dimeric RG-II (dRG-II) was included, obtained from *Arabidopsis* leaves through digestion with endo-polygalacturonase. The monomeric form of RG-II (mRG-II) was generated by acid treatment of the dimer. (B) The figure shows a re-dimerisation assay used to confirm that the isolated domain corresponds to RG-II. To achieve this, the dimeric form was first monomerised via acidic treatment, then subjected to re-dimerisation, as described by [6]. Lanes: 1) 1.6 μg of dRG-II; 2) 1.6 μg of mRG-II (dRG-II treated with HCl); 3) 1.6 μg of mRG-II; 4) 1.6 μg of mRG-II + Pb^2+^ + H_3_BO_3_; 5) 1.6 μg of mRG-II + Pb^2+^; 6) 1.6 μg of mRG-II + H_3_BO_3_; 7) 2.4 μg each of dimeric and monomeric RG-II prepared from *Arabidopsis* leaves (used as markers). Reactions 3–6 were prepared in a final volume of 10 µL using 50 mM sodium acetate buffer (pH 4.8), 0.5 mM Pb(NO_3_)_2_, and 1.2 mM H_3_BO_3_. All samples were incubated overnight at room temperature.

The protocol described here enables, for the first time, the isolation of RG-II from *Arabidopsis* seed mucilage using a limited quantity of seeds as starting material. Owing to the characteristics of the selected column, it is possible to separate RG-II from other pectic components, such as RG-I and oligogalacturonides (Figure 2A). The identity of RG-II was confirmed through a monomerisation/re-dimerisation assay (Figure 2B). The results presented in Table 2 show that the method is consistent, yielding similar amounts of isolated RG-II regardless of whether wild-type or mutant seeds are used. Given the complex structure of this pectic domain, the ability to isolate it opens the door to further studies, for instance, investigating mutant lines with defects in mucilage synthesis, structure, or release. Such studies may have previously overlooked the potential involvement of RG-II due to the lack of suitable tools. This method now offers a reliable means of isolating RG-II, facilitating its characterisation using techniques such as PAGE, HPLC, FTIR, or MALDI-TOF MS.

**Table 2.** Yield of isolated RG-II

Genotype	Seed (mg)	Total GalA (µg)	mg GalA/g seed
Col-0 WT	49.73	6.31	0.13
*bxl 1.1*	55.70	5.62	0.10
*bxl 1.2*	52.17	6.54	0.13
*bxl 1. 3*	51.83	6.08	0.12

## General notes and troubleshooting

**Troubleshooting**

1. Ensure that the solution is properly neutralised after saponification, as insufficient neutralisation may affect the efficiency of digestion with endo-polygalacturonase.

2. Any residual carbonate in the digestion solution may cause bubbling when it comes into contact with the acetic acid present in the column running buffer. This can damage the column by altering its separation capacity.

3. Make sure that the water used to prepare the gel and solutions for its development is free of chloride ions (Cl-), as these may precipitate in the presence of silver ions (Ag^+^).

## Validation of protocol

This protocol was used for the detection and isolation of RG-II from *Vasconcellea pubescens* seed mucilage (Sanhueza et al. [8] https://doi.org/10.3389/fpls.2024.1380533). It was also applied for the isolation of RG-I from *Arabidopsis* seed mucilage (Saez-Aguayo et al. [9] https://doi.org/10.1016/j.tcsw.2024.100134).
